# Pre-ischemic Lactate Levels Affect Post-ischemic Recovery in an Isolated Rat Heart Model of Donation After Circulatory Death (DCD)

**DOI:** 10.3389/fcvm.2021.669205

**Published:** 2021-06-14

**Authors:** Maria Arnold, Adrian Segiser, Selianne Graf, Natalia Méndez-Carmona, Maria N. Sanz, Rahel K. Wyss, Nina Kalbermatter, Nino Keller, Thierry Carrel, Sarah Longnus

**Affiliations:** ^1^Department of Cardiovascular Surgery, Inselspital, Bern University Hospital, Bern, Switzerland; ^2^Department for BioMedical Research, University of Bern, Bern, Switzerland

**Keywords:** cardiac ischemia reperfusion injury, donation after circulatory death, heart transplantation, lactate, contractile function

## Abstract

**Introduction:** Donation after circulatory death (DCD) could substantially improve donor heart availability. In DCD, the heart is not only exposed to a period of warm ischemia, but also to a damaging pre-ischemic phase. We hypothesized that the DCD-relevant pre-ischemic lactate levels negatively affect the post-ischemic functional and mitochondrial recovery in an isolated rat heart model of DCD.

**Methods:** Isolated, working rat hearts underwent 28.5′ of global ischemia and 60′ of reperfusion. Prior to ischemia, hearts were perfused with one of three pre-ischemic lactate levels: no lactate (0 Lac), physiologic lactate (0.5 mM; 0.5 Lac), or DCD-relevant lactate (1 mM; 1 Lac). In a fourth group, an inhibitor of the mitochondrial calcium uniporter was added in reperfusion to 1 Lac hearts (1 Lac + Ru360).

**Results:** During reperfusion, left ventricular work (heart rate-developed pressure product) was significantly greater in 0.5 Lac hearts compared to 0 Lac or 1 Lac. In 1 vs. 0.5 Lac hearts, in parallel with a decreased function, cellular and mitochondrial damage was greater, tissue calcium content tended to increase, while oxidative stress damage tended to decrease. The addition of Ru360 to 1 Lac hearts partially abrogated the negative effects of the DCD-relevant pre-ischemic lactate levels (greater post-ischemic left ventricular work and less cytochrome c release in 1 Lac+Ru360 vs. 1 Lac).

**Conclusion:** DCD-relevant levels of pre-ischemic lactate (1 mM) reduce contractile, cellular, and mitochondrial recovery during reperfusion compared to physiologic lactate levels. Inhibition of mitochondrial calcium uptake during early reperfusion improves the post-ischemic recovery of 1 Lac hearts, indicating calcium overload as a potential therapeutic reperfusion target for DCD hearts.

## Introduction

Donation after circulatory death (DCD) as an alternative source of cardiac grafts for transplantation could substantially improve the shortage of donor organs. Indeed, more than 140 DCD heart transplantations have been performed to date (1–4) with patient outcomes similar to those with conventional donation after brain death at one- and five-year time points (1, 4, 5). The use of DCD heart transplantation has permitted an increase in adult heart transplantations by 15% in one Australian DCD center (1) and even 48% in one Cambridge, UK DCD center (4). In light of these promising results, it is critical to identify and develop optimal clinical protocols to ensure the safe and effective adoption of DCD heart transplantation.

Conditions surrounding circulatory arrest can affect the quality of cardiac grafts. Currently, only donors from the Maastricht category III are used for DCD cardiac donation (3, 5–7). With this type of donor, cardiac arrest can be anticipated as it includes patients that are already in the hospital and the family has agreed to withdraw life-sustaining therapy (WLST). This category is also considered as a controlled DCD because the circumstances and time of ischemia are known and kept to a minimum (8). Importantly, prior to procurement, DCD cardiac grafts are exposed not only to a deleterious period of warm ischemia, but also to a potentially damaging pre-ischemic phase after WLST (9, 10). Acute, high levels of circulating free fatty acids are expected in Maastricht category III DCD donors. For example, in patients undergoing heart surgery, high plasma levels of free fatty acids (1.6–2.2 mmol/L) were observed (11) as well as in patients with myocardial infarction (12). These elevations of circulating free fatty acids could result from the increased catecholamine levels (12, 13) and/or heparin administration (14). Furthermore, markedly increased catecholamine levels prior to cardiac arrest have been observed in porcine DCD models (9, 10). In addition, in an *in-situ* rat model of DCD, we measured free fatty acids concentrations of 1.22 ± 0.69 mmol/L at the onset of functional warm ischemia (15). We previously reported that an acute, pre-ischemic exposure to high levels of palmitate significantly lowered the post-ischemic cardiac function in an isolated rat heart model of DCD (16), indicating an important role of substrate availability prior to ischemia. Lactate also plays an important role in cardiac metabolism, but the role of increased lactate levels before cardiac arrest has not yet been investigated. After WLST, organs are subjected to a period of hypoxia prior to the start of functional warm ischemia, which leads to increased levels of circulating lactate. In an *in-situ* rat model of DCD, we recently reported arterial lactate levels of 1.05 ± 0.46 mmol/L at the onset of functional warm ischemia (when systolic arterial pressure dropped to 50 mmHg) (15). Only a few studies have investigated the effects of pre-ischemic lactate levels on heart recovery and these studies used much higher levels of lactate (17, 18). For example, Goodwin et al. reported an improved post-ischemic recovery of cardiac power in hearts perfused with 10 mM lactate and 11 mM glucose before ischemia compared to glucose only hearts (18).

Lactate may be important in cardiac adaptation to ischemia-reperfusion injury. It has long been regarded as a metabolic waste product with deleterious effects (19). Nowadays, lactate is recognized as a link between aerobic and glycolytic pathways, being the product of one metabolic pathway (glycolysis) and the substrate for another (oxidative phosphorylation) (20). Not only does lactate play a role as a major energy source or as a gluconeogenic precursor, but it also acts as a signaling molecule (20). Importantly, lactate and hypoxia-inducible factor-1 (HIF-1), the master regulator of oxygen homeostasis, are regulated by reciprocal activation, i.e., increased lactate levels promote HIF-1 stabilization and expression of HIF-1-induced genes, while HIF-1 stimulates glucose uptake and glycolysis, and thereby, lactate production (19, 21, 22). A similar relationship of reciprocal activation exists between lactate and peroxisome proliferator activated receptor gamma coactivator-1 alpha (PGC-1α), the master regulator of mitochondrial biogenesis (21, 23). Although lactate may play an important role in cardiac adaptation to ischemia-reperfusion injury, the role of lactate signaling specifically in heart muscle in response to ischemia and reperfusion has not yet been investigated.

Calcium overload, together with a burst of reactive oxygen species and a rapid pH normalization, is one of the key mediators of lethal reperfusion injury (24). Intracellular and mitochondrial calcium overload, which begins during ischemia, is exacerbated at the time of reperfusion due to the oxidative stress-induced dysfunction of the sarcoplasmic reticulum, plasma membrane disruption, and mitochondrial re-energization (25). Excess calcium induces cardiomyocyte death by causing hypercontracture and opening of the mitochondrial permeability transition pore (mPTP) (24). Studies in isolated rat hearts have shown a protective effect of an oxygen-bridged dinuclear ruthenium amine complex (Ru360), a specific mitochondrial calcium uniporter (MCU) inhibitor, through the prevention of mPTP opening (26). Although lactate has been shown in other tissues to induce intracellular calcium elevations that stimulated plasticity-related gene expression (27), the role of pre-ischemic lactate levels on calcium overload in myocardial reperfusion is still unclear.

Importantly, interventions prior to graft procurement are limited for legal and ethical reasons in DCD donors. However, systemic changes surrounding the donor graft can influence the graft's ischemic tolerance. Given that DCD cardiac grafts are exposed to increased circulating lactate prior to warm ischemia, we hypothesized that these pre-ischemic lactate levels negatively affect the post-ischemic functional and mitochondrial recovery in an isolated rat heart model of DCD. Furthermore, we investigated whether prevention of mitochondrial calcium overload at early reperfusion abolishes the negative effects of DCD-relevant pre-ischemic lactate levels on post-ischemic cardiac recovery. To do so, we inhibited the mitochondrial calcium uniporter with Ru360.

## Materials and Methods

### Ethics Statement

All experimental procedures were carried out in compliance with the European Convention for Animal Care and approved by the Swiss animal welfare authorities and the Ethics Committee for Animal Experimentation, Berne, Switzerland. Surgery was performed under general anesthesia, and all efforts were made to minimize animal suffering.

### Isolated Heart Perfusions

A well-established isolated rat heart model of DCD was used (16, 28–31). Male Wistar rats (Janvier Labs, Le Genest-Saint-Isle, France) aged 11–12 weeks were used to ensure mature cardiac metabolism (32) and to represent young, adult human DCD donors. Rats were housed under standard conditions with *ad libitum* access to water and food, a 12 h light-dark cycle, and a controlled room temperature. After random assignment to experimental groups, rats were anesthetized intraperitoneally with 100 mg/kg of ketamine (Narketan®, Vetoquinol AG, Bern, Switzerland) and 10 mg/kg of xylazine (Xylapan®, Vetoquinol AG, Bern, Switzerland). As soon as the pedal reflex had disappeared, hearts were explanted and cannulated on the perfusion system. For the first 20 min, hearts underwent an aerobic working mode perfusion with a modified Krebs-Henseleit bicarbonate (KHB) buffer (containing 118 mM NaCl, 4.7 mM KCl, 1.2 mM KH_2_PO_4_, 1.25 mM CaCl_2_•2H_2_O, 1.2 mM MgSO_4_•7H_2_O, 25 mM NaHCO_3_, and 11 mM glucose, supplemented with 1.2 mM palmitate/3% BSA; gassed with 95% O_2_-5% CO_2_). Hearts were spontaneously beating and maintained at 37°C throughout the perfusion protocol.

**Figure 1:**
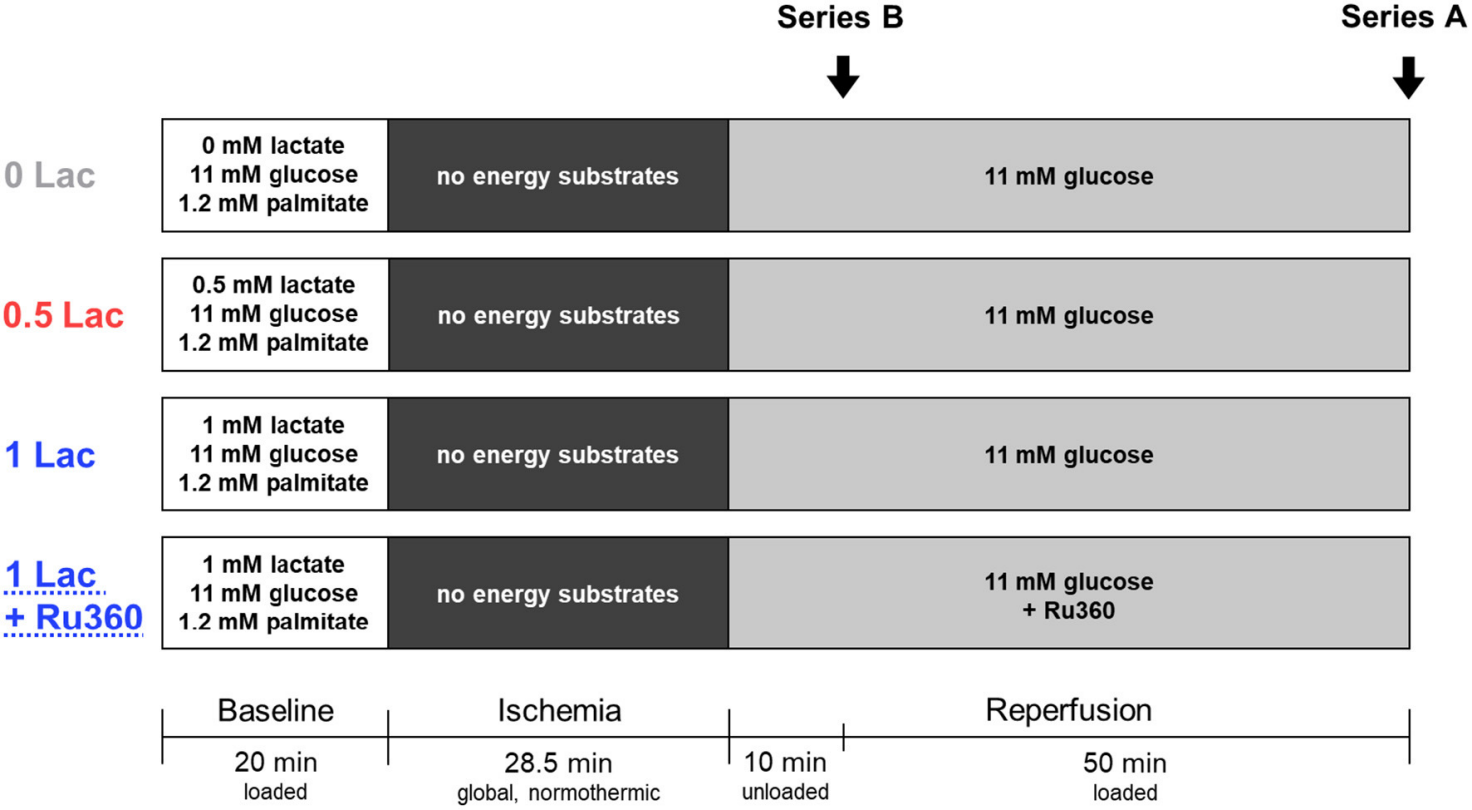
Experimental protocol. Series A: Hearts underwent 20 min of baseline working-mode (loaded) perfusion, followed by 28.5 min of warm, global ischemia, and 60 min of reperfusion [10 min unloaded followed by 50 min working-mode (loaded)]. In addition to 11 mM glucose and 1.2 mM palmitate, three pre-ischemic (baseline) lactate levels were tested: no lactate (0 Lac), physiologic lactate (0.5 mM; 0.5 Lac), or DCD-relevant lactate (1 mM; 1 Lac). In a fourth group, hearts with DCD-relevant pre-ischemic lactate levels were treated with a mitochondrial calcium uniporter inhibitor, Ru360, during reperfusion (1 Lac + Ru360). Series B: The same four experimental groups were used, but perfusions were stopped after 10 min of reperfusion in order to isolate mitochondria.

Three different energy-substrate buffer compositions during baseline were tested: either no lactate (0 Lac; *n* = 9), physiologic lactate (0.5 mM sodium L-lactate; 0.5 Lac; *n* = 14), or DCD-relevant lactate (1 mM sodium L-lactate; 1 Lac; *n* = 7) were included in addition to 11 mM glucose and 1.2 mM palmitate. Thereafter, all hearts were subjected to 28.5 min of global, no-flow, normothermic (37°C) ischemia. An ischemic duration of 28.5 min was chosen as it is clinically relevant for DCD, and because this duration provides an intermediate level of post-ischemic functional recovery, permitting the determination of either increases or decreases in response to the experimental conditions/applied treatments. The first 10 min of reperfusion following ischemia were performed in an unloaded mode (according to the standard clinical practice in cardiac surgery); subsequently, hearts were switched to a working mode for the following 50 min of reperfusion (same reperfusion buffer for all groups: modified KHB buffer without the addition of palmitate/BSA and lactate). Additionally, in a fourth experimental group, hearts with DCD-relevant pre-ischemic lactate levels (1 mM) were treated with an MCU inhibitor, Ru360 (250 nmol/L, Sigma-Aldrich, St. Louis, MO, USA; 1 Lac + Ru360; *n* = 7), during the reperfusion period (Figure 1, Series A).

As soon as the perfusion protocol ended, perfusate lines to the heart were clamped and the heart was immediately frozen with liquid nitrogen-cooled Wollenberger clamps. Afterwards, the heart was put into liquid nitrogen and stored at −80°C until needed.

In a second series of hearts (Figure 1, Series B), the same four experimental groups (0 Lac, *n* = 7; 0.5 Lac, *n* = 8; 1 Lac, *n* = 7; 1 Lac + Ru360, *n* = 6) were generated according to the above-mentioned perfusion protocol, except perfusions were stopped after 10 min of reperfusion in order to freshly isolate mitochondria from ventricular tissue.

Buffer samples from preload line and coronary effluent were taken throughout the perfusion protocol and stored at −80°C until analysis.

### Functional Data Collection

A micro-tip pressure catheter (Millar, Houston, TX, USA), placed in the left ventricle, was used to continuously measure the peak systolic pressure, developed pressure, minimum and maximum first derivatives of left ventricular pressure (dP/dt min and dP/dt max), and heart rate. Left ventricular work (LV work) was calculated as the product of developed pressure and heart rate.

Flowmeters (Transonic Systems Inc., Ithaca, NY, USA), placed in preload and afterload lines, were used to determine the cardiac output and coronary flow.

All data were recorded using the PowerLab data acquisition system (ADInstruments, Spechbach, Germany).

### Cardiac Oxygen Consumption and Oxygen Efficiency

With the help of a blood-gas analyzer (Cobas b 123; Roche, Basel, Switzerland), oxygen consumption was determined. Cardiac oxygen consumption was calculated as:

$$\begin{array}{l} \frac{\left(C_{PL}\left(t\right)\;-\;C_{CE}\left(t\right)\right)\;\times \;CF\left(t\right)}{HW} \end{array}$$

Cardiac oxygen efficiency was calculated as:

$$\begin{array}{l} \frac{LV\;work\;\left(t\right)}{O_{2}C\;\left(t\right)} \end{array}$$

C = concentration, CE = coronary effluent, CF = coronary flow, HW = heart weight, LV work = left ventricular work, O_2_C = oxygen consumption, PL = preload, t = time point of interest.

### Lactate

Buffer samples from 0 and 20 min baseline and 0, 5, and 60 min reperfusion time points were used for lactate measurements using a commercially available kit (Sigma-Aldrich, St. Louis, MO, USA). Lactate accumulation was calculated as:

$$\frac{\left(C_{t}\;\times \;V_{t})-(C_{0\;min}\;\times \;V_{0\;min}\right)}{HW}$$

C = concentration in recirculating buffer, HW = heart weight, t = time point of interest, V = buffer volume.

### Markers/Indicators of Cell Death and Mitochondrial Damage

Buffer samples from 10 and 60 min of reperfusion were used to measure the markers of cell death and mitochondrial damage. For the markers of cell death, the Rat Myoglobin ELISA (Life Diagnostics, West Chester, PA, USA) and Rat heart-type fatty acid binding protein (H-FABP) ELISA (Life Diagnostics, West Chester, PA, USA) were used. For mitochondrial damage, cytochrome c release was determined with the Quantikine ELISA kit for Rat/Mouse Cytochrome c (R&D Systems, Minneapolis, MN, USA). Release of all markers was calculated as:

$$\frac{\left(C_{60\;\min}\;\times \;V_{60\;\min})-(C_{10\;\min}\;\times \;V_{10\;\min}\right)}{50\min\;\times \;HW}$$

C = concentration in recirculating buffer, HW = heart weight, V = buffer volume.

### mRNA Expression

mRNA expression was measured according to the protocol described earlier (30). RPLP0 and Ywhaz were selected as the reference genes. The set limit for replicate variability was 0.7 cycles. The used primer sequences are presented in Table 1.

**Table 1:** Sequences of the F (forward) and R (reverse) primers used for the following genes: MCT1 (Slc16a1), monocarboxylate transporter 1; PGC-1α, peroxisome proliferator-activated receptor gamma coactivator 1-α; RPLP0, ribosomal protein lateral stalk subunit P0; Ywhaz, tyrosine 3-monooxygenase/tryptophan 5-monooxygenase activation protein.

**Gene**	**Primers**
PGC-1α (NM_031347.1)	F: 5′- GTG GAT GAA GAC GGA TTG CC−3′
	R: 5′- GGT GTG GTT TGC ATG GTT CT−3′
MCT1 or Slc16a1 (NM_012716.2)	F: 5′- GCG CCG CGA GAT ACA CAT A−3′
	R: 5′- CAC TTC ACT GGT CGT TGC AC−3′
Ywhaz (NM_013011)	F: 5′- AGA CGG AAG GTG CTG AGA AA−3′
	R: 5′- GAA GCA TTG GGG ATC AAG AA−3′
RPLP0 (NM_022402)	F: 5′- GCG ACC TGG AAG TCC AAC TA−3′
	R: 5′- TTG TCT GCT CCC ACA ATG AA−3′

### Markers of Oxidative Stress

Protein carbonylation in total tissue was detected with the OxyBlotTM Protein Oxidation Detection kit (Merck Millipore, Burlington, MA, USA), as previously described (28). Small changes to the kit's protocol were made: a fluorescent secondary antibody was used (goat anti-rabbit; Invitrogen, Carlsbad, CA, USA), and GAPDH (primary antibody (Santa Cruz Biotechnology, Dallas, TX, USA) was used as the loading control: 1:1,000 in the PBS-Odyssey Blocking Buffer (LI-COR Biosciences, Lincoln, NE, USA); secondary antibody (goat anti-mouse; LI-COR Biosciences, Lincoln, NE, USA): 1:10,000 in the PBS-Odyssey Blocking Buffer (LI-COR Biosciences, Lincoln, NE, USA) (Supplementary Figure 1).

### Tissue Calcium Content

Total tissue calcium content was determined with the Colorimetric Calcium Detection Assay kit (Abcam, Cambridge, UK) according to the manufacturer's instructions. As a control and for method validation, calcium tissue content was measured in a comparable series of non-ischemic control hearts that were previously perfused for another study (33).

### Isolation and Quantification of Cardiac Mitochondria

Cardiac mitochondria were isolated as previously described (28), and the pelleted mitochondria were resuspended in 0.3 mL of buffer (70 mmol/L sucrose, 210 mmol/L mannitol, 50 mmol/L Tris-HCl, 0.1 mmol/L EDTA) for immediate analysis. For an indication of the mitochondrial mass, protein content of the isolated, fresh mitochondria was measured with the bicinchoninic acid (BCA) assay kit (Thermo Fisher Scientific, Massachusetts, USA). Freshly isolated mitochondria were then further analyzed with a calcium retention capacity assay (see below), and the remaining mitochondria suspension was aliquoted and frozen at −80°C for later determination of citrate synthase activity and calcium content. Citrate synthase activity of the isolated, frozen mitochondria was measured using a standard spectrophotometric assay, as previously described (28, 34).

### Mitochondrial Calcium Retention Capacity

The mitochondrial calcium retention capacity was assessed in freshly isolated mitochondria, as previously described (28, 35, 36), with some modifications. The assay was adapted for a 96-well plate and measured with a Tecan plate reader (Tecan Life Sciences, Männedorf, CH). Briefly, freshly isolated mitochondria (48 μg of protein) were dissolved in a buffer containing 150 mmol/L sucrose, 2 mmol/L KH_2_PO_4_, 20 mmol/L Tris-HCl, 50 mmol/L KCl, 10 mmol/L glutamate, 4 mmol/L malate, pH 7.4. An extra-mitochondrial, calcium-sensitive, fluorescent probe (Calcium Green 5N, Invitrogen, Carlsbad, CA; 0.5 umol/L; ex. 485 nm, em. 532 nm) was used to observe the mitochondrial calcium uptake, and a sudden release (increase of fluorescent signal) was associated with the mPTP opening. After a baseline measurement of 3 min, the reaction was initiated through the addition of one single calcium spike (either 0.1, 0.3, or 0.5 mmol/L CaCl_2_) and fluorescence was monitored (Table 2, Supplementary Figure 2). At the end of the assay, 0.1 mmol/L carbonyl cyanide 3-chlorophenylhydrazone (CCCP, Sigma-Aldrich, St. Louis, Missouri, USA) was added as a positive control to permeabilize the inner mitochondrial membrane and, therefore, induce calcium release from any remaining intact mitochondria. To delay and, therefore, better observe the mitochondrial calcium uptake and subsequent mPTP opening, ADP was added to some mixes (37). As a positive control, 1 μmol/L cyclosporine A (CsA, Sigma-Aldrich, St. Louis, Missouri, USA), an inhibitor of the mPTP opening, was added to some mixes. As a negative control, some mixes were measured without calcium additions. Furthermore, for method validation, mitochondria were isolated from four non-ischemic hearts (Langendorff perfusion for 1 h) and subjected to the assay.

**Table 2:** Additions to the various solutions used for the calcium retention capacity assay.

	**Mix 1**				**Mix 2**				**Mix 3**		**Mix 4**	
ADP					x	x	x	x			x	x
CsA									x	x	x	x
No calcium				x				x		x		x
0.1 mM CaCl_2_	x				x							
0.3 mM CaCl_2_		x				x						
0.5 mM CaCl_2_			x				x		x		x	

*For each mix, isolated mitochondria were maintained in a buffer containing 150 mmol/L sucrose, 2 mmol/L KH_2_PO_4_, 20 mmol/L Tris-HCl, 50 mmol/L KCl, 10 mmol/L glutamate, 4 mmol/L malate. Furthermore, a calcium-sensitive, fluorescent probe (Calcium Green 5N) was added to each mix. CsA, cyclosporine A*.

### Mitochondrial Calcium Content

Mitochondrial calcium content was measured in the frozen, isolated mitochondria as previously described (28). Briefly, mitochondria (7 mg/mL protein content) were lysed with 0.1 mmol/L Triton X-100 and then added to a solution of 150 mmol/L sucrose, 2 mmol/L KH_2_PO_4_, 20 mmol/L Tris-HCl, and 50 mmol/L KCl (pH 7.4). With 0.2 μmol/L Calcium Green 5N (Invitrogen, Carlsbad, CA, USA), a calcium-sensitive probe, samples were measured fluorometrically, and calcium content was normalized to citrate synthase activity in the respective samples.

### Statistical Analysis

All statistical analyses were conducted with the GraphPad Prism 8 (GraphPad Software, Inc., La Jolla, CA, USA). Outliers were detected with the Tukey's box and whiskers plot and removed from the statistical analyses (38, 39). Spearman rank order correlation was used to evaluate relationships between the pre-ischemic lactate levels and different post-ischemic outcome measurements (mRNA expression, protein carbonylation). For an overview of the differences between the experimental groups, Kruskal-Wallis tests were carried out. When significant overall results were observed, comparisons between groups were performed using the Mann-Whitney U tests. The *p*-values were all two-tailed and adjusted for multiple comparisons (modified sequential rejective Bonferroni procedure) (40). Corrected *p*-values are reported and considered statistically significant if <0.05. All values are expressed as mean ± standard deviation or as median, 25–75 percentiles, and range (box-and-whiskers).

## Results

### Cardiac Functional Performance

**Figure 2:**
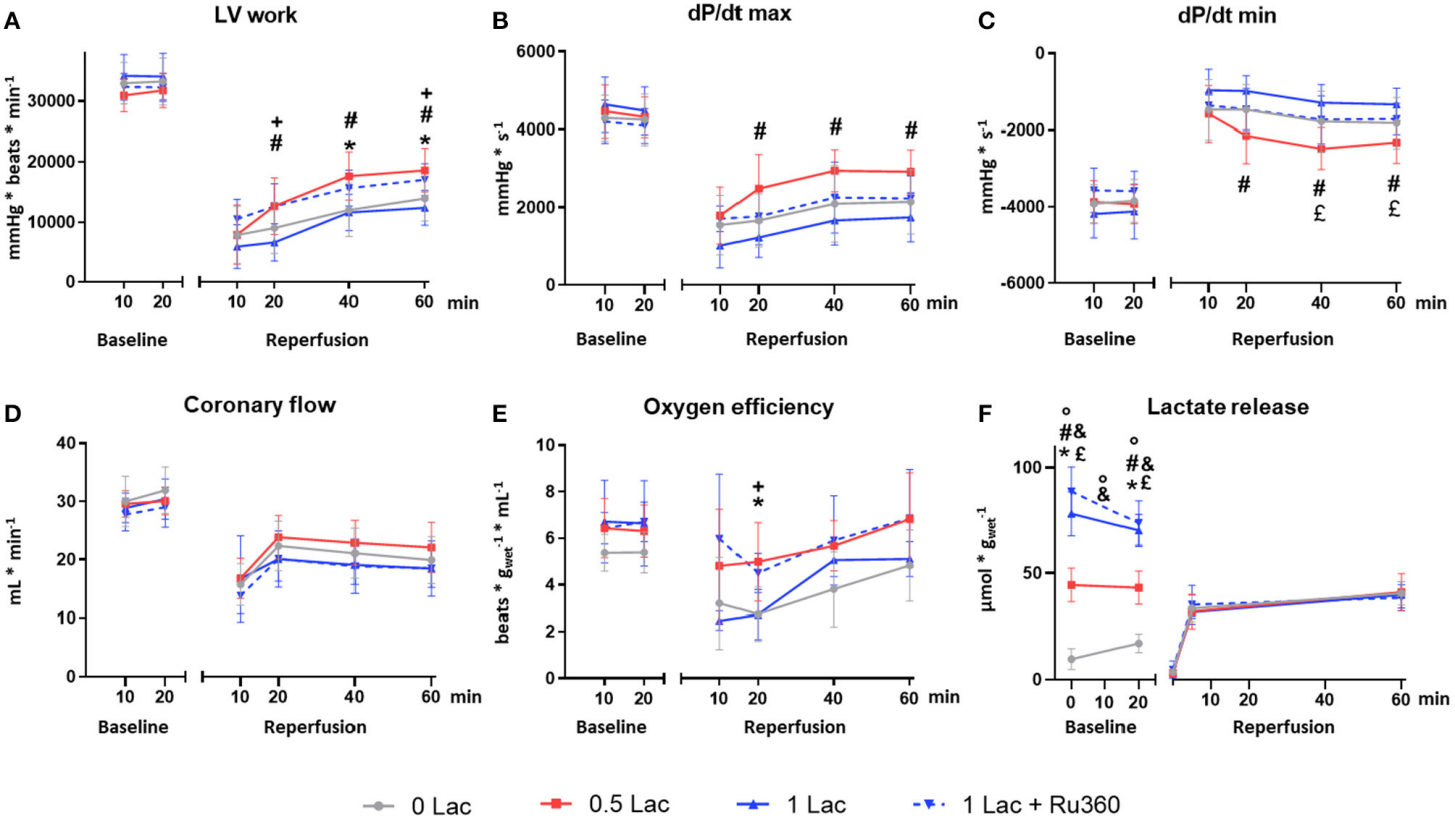
Functional and metabolic parameters. **(A)** Left ventricular work (LV work = developed pressure*heart rate) **(B)** dP/dt max (maximum first derivative of LV pressure) **(C)** dP/dt min (minimum first derivative of LV pressure) **(D)** Coronary flow **(E)** Cardiac oxygen efficiency [LV work/oxygen consumption] **(F)** Lactate release. *p < 0.05 for 0 vs. 0.5 Lac; #*p* < 0.05 for 0.5 vs. 1 Lac; °*p* < 0.05 for 0 vs. 1 Lac; +*p* < 0.05 for 1 Lac vs. 1 Lac + Ru360; £ *p* < 0.05 for 0.5 Lac vs. 1 Lac + Ru360; &*p* < 0.05 for 0 Lac vs. 1 Lac + Ru360. *n* = 7–14/group. Data are expressed as mean ± standard deviation.

During baseline perfusion, no difference was observed for any functional parameter among the experimental groups (Figures 2A–D).

After ischemia, LV work was significantly greater in 0.5 Lac hearts compared to 0 Lac or 1 Lac (*p* < 0.05 for both at 40 and 60 min of reperfusion; Figure 2A). Furthermore, LV work in 1 Lac + Ru360 hearts was significantly higher compared to 1 Lac hearts (*p* < 0.05 at 20 and 60 min of reperfusion; Figure 2A), and did not differ compared to 0.5 Lac hearts. Similar patterns between 1 Lac vs. 0.5 Lac hearts were observed for post-ischemic contraction (dP/dt max) and relaxation (dP/dt min) rates, with significant differences at 20, 40, and 60 min of reperfusion (*p* < 0.05 for all; Figures 2B,C). However, 1 Lac + Ru360 hearts presented the same post-ischemic dP/dt max and dP/dt min as 1 Lac hearts (Figures 2B,C), despite the differing LV work results. In late reperfusion (20, 40, 60 min), no differences between the experimental groups were observed for the coronary flow (Figure 2D) or cardiac output, which ranged from 0 to 3 mL/min (data not shown). A tendency toward a greater oxygen efficiency during reperfusion in 0.5 Lac and 1 Lac + Ru360 vs. 0 Lac or 1 Lac hearts was observed but reached statistical significance only at 20 min of reperfusion in 0.5 Lac vs. 0 Lac hearts and 1 Lac + Ru360 vs. 1 Lac hearts (*p* < 0.05; Figure 2E).

### Lactate Release

The lactate measured in the buffer during baseline was, in correspondence with the experimental protocol, lowest in 0 Lac and highest in 1 Lac and 1 Lac + Ru360 hearts (*p* < 0.05 among all groups except 1 Lac vs. 1 Lac + Ru360 at 0 and 20 min baseline; Figure 2F). For 0 Lac hearts, a net lactate release was measured from 0 to 20 min baseline (*p* < 0.05 0 vs. 20 min baseline), whereas in 0.5 Lac, lactate values did not differ between 0 and 20 min baseline and in 1 Lac and 1 Lac + Ru360 hearts, a non-significant tendency for a net lactate use during baseline was observed. The net rate of change (slope between 0 and 20 min baseline) was significantly different in 0 vs. 1 Lac and 1 Lac + Ru360 hearts (*p* < 0.05 for both).

During reperfusion, no exogenous lactate was added to the buffer and this was reflected in the measurement at 0 min of reperfusion for all the groups. During reperfusion, a similar net lactate release was observed in all experimental groups.

### Cell Death

**Figure 3:**
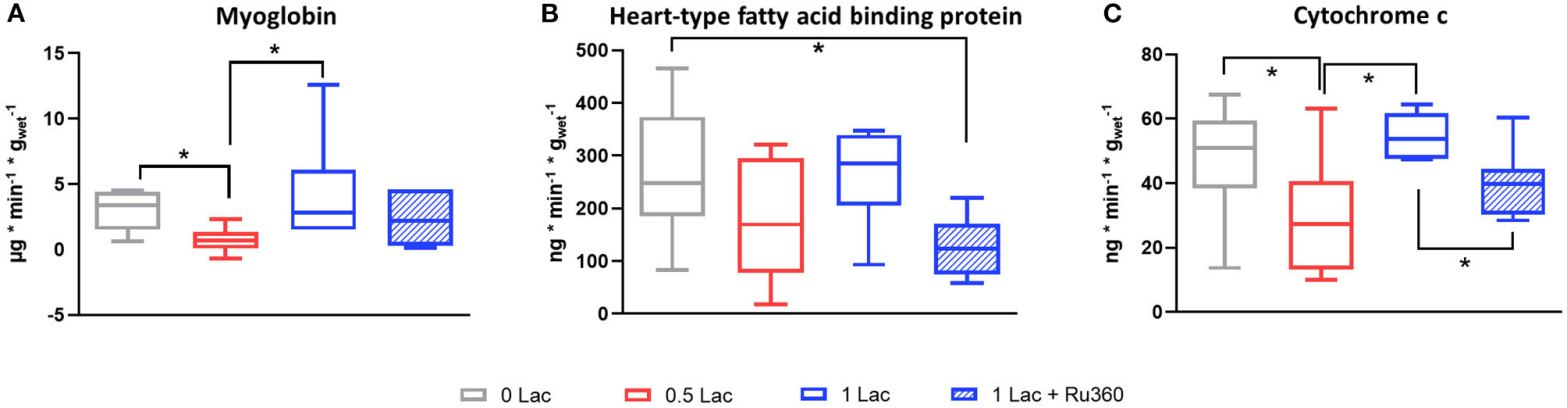
Release of circulating markers of cell death and mitochondrial damage between 10 and 60 min of reperfusion. **(A)** Myoglobin **(B)** Heart-type fatty acid binding protein (H-FABP) **(C)** Cytochrome c (cyt c). *p < 0.05; *n* = 6–14/group. Data are expressed as median, 25–75 percentiles, and range.

Release of the cell death marker myoglobin was significantly lower in 0.5 Lac hearts compared to 0 Lac or 1 Lac hearts (*p* < 0.05 for both), but not different compared to 1 Lac + Ru360 hearts (Figure 3A). H-FABP release was significantly lower in 1 Lac + Ru360 compared to 0 Lac hearts (*p* < 0.05), whereas 1 Lac and 0 Lac hearts did not differ in terms of H-FABP release (Figure 3B).

### Mitochondrial Damage

Less cytochrome c (mitochondrial damage marker) was released in 0.5 vs. 0 Lac and 1 Lac hearts (*p* < 0.05 for both; Figure 3C). Additionally, 1 Lac + Ru360 hearts presented a lower cytochrome c release compared to 1 Lac hearts (*p* < 0.05; Figure 3C).

### mRNA Expression of PGC-1α and MCT1

**Figure 4:**
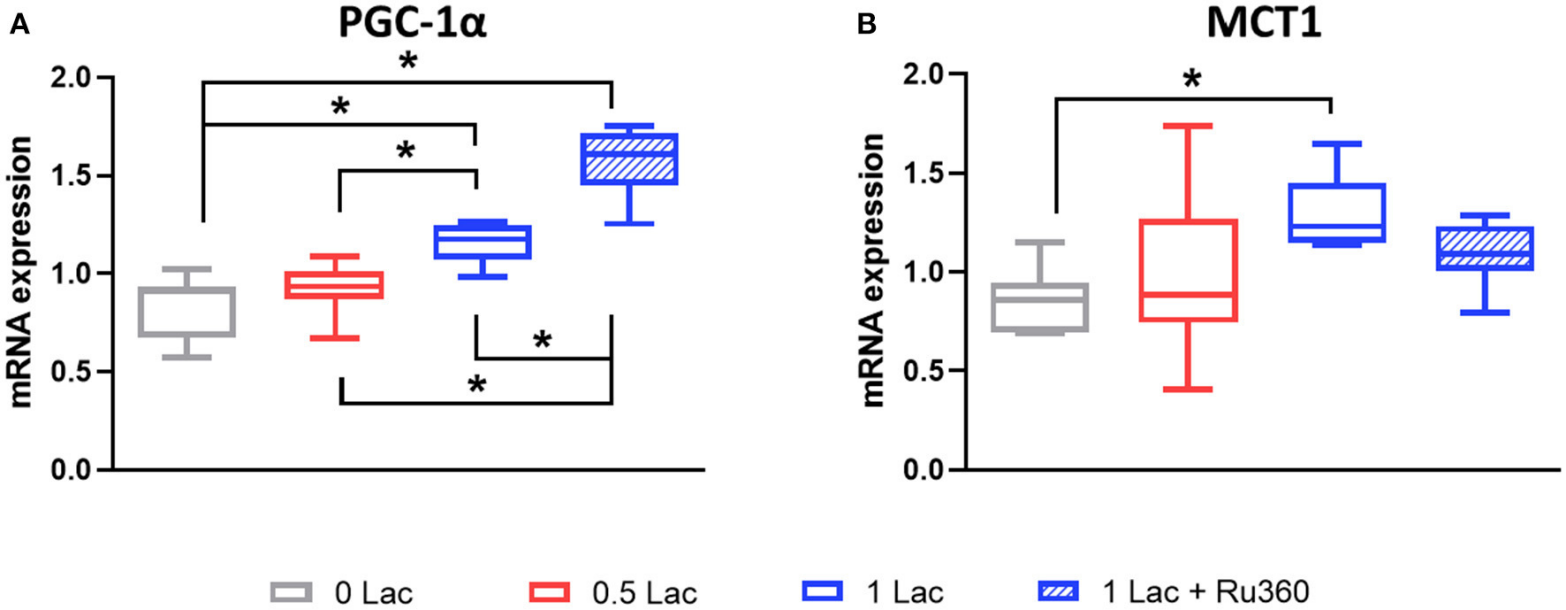
mRNA expressions after 60 min of reperfusion. **(A)** PGC-1α, peroxisome proliferator-activated receptor γ coactivator-1 α **(B)** MCT1, monocarboxylate transporter 1 (Slc16a1). *p < 0.05; *n* = 5–11/group. Data are expressed as median, 25–75 percentiles, and range.

A total of 28.5 min of global, warm ischemia followed by 60 min of reperfusion significantly modified the mRNA expressions of PGC-1α, a master regulator of mitochondrial biogenesis, and monocarboxylate transporter 1 (MCT1), a lactate transporter, among the different experimental groups (*p* < 0.05 all comparisons except 0 vs. 0.5 Lac for PGC-1α, 0 vs. 1 Lac for MCT1; Figure 4). Furthermore, pre-ischemic lactate levels correlated positively with PGC-1α and MCT1 mRNA expressions at 60 min of reperfusion (Spearman correlation *p* < 0.05 for both for 0 Lac and 0.5 Lac and 1 Lac hearts; not shown).

### Oxidative Stress

**Figure 5:**
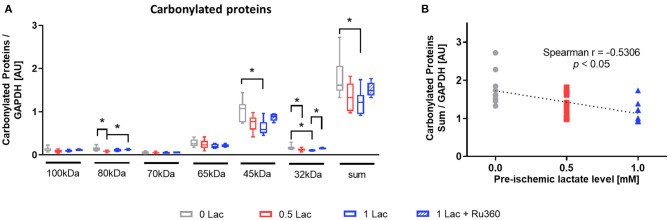
Marker of oxidative stress damage. **(A)** Protein carbonylation measured by oxyblot at 60 min of reperfusion **(B)** Spearman correlation of pre-ischemic lactate level with carbonylated proteins (sum) at 60 min of reperfusion. *p < 0.05; *n* = 9–14/group. Data are expressed as median, 25–75 percentiles, and range.

The level of carbonylated proteins was highest in 0 Lac hearts (*p* < 0.05 0 Lac vs. 0.5 Lac for 80 kDa and 32 kDa, as well as *p* < 0.05 0 Lac vs. 1 Lac for 45 kDa, 32 kDa, and the sum; Figure 5A). Furthermore, the amount of carbonylated proteins after 60 min of reperfusion correlated negatively with the pre-ischemic lactate levels (Spearman correlation *p* < 0.05 for 0 Lac, 0.5 Lac, and 1 Lac hearts; Figure 5B).

### Calcium Content

**Figure 6:**
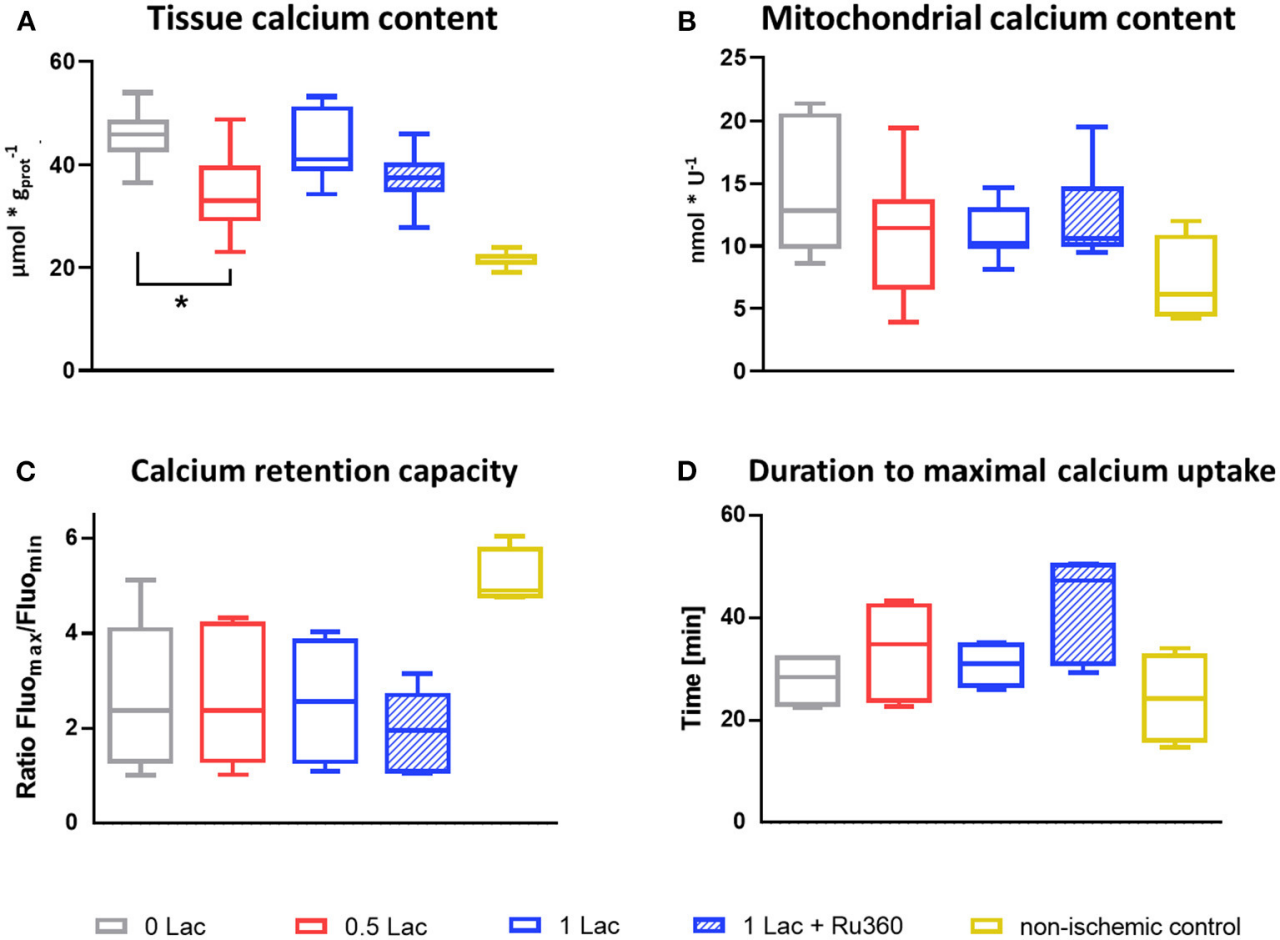
Calcium content and mitochondrial calcium retention capacity. **(A)** Cardiac tissue calcium content at 60 min of reperfusion, *n* = 6–14/group, non-ischemic controls (*n* = 7) are historical data from the lab (no stats performed) **(B)** Mitochondrial calcium content at 10 min of reperfusion, *n* = 6–8/group, except non-ischemic controls *n* = 4 **(C)** Mitochondrial calcium retention capacity (measured in freshly isolated mitochondria after 10 min of reperfusion) expressed as ratio of the maximal and minimal fluorescence [AU] after the addition of 0.3 mM CaCl_2_, Mix 2. **(D)** Time between CaCl_2_ addition (0.3 mM, Mix 2) until minimal fluorescence value (=duration to maximal calcium uptake by mitochondria). *p < 0.05; *n* = 4–6/group, except non-ischemic controls *n* = 4 **(C,D)**. Data are expressed as median, 25–75 percentiles, and range.

The 0.5 Lac hearts had a lower total tissue calcium content than 0 Lac hearts (*p* < 0.05; Figure 6A) at 60 min of reperfusion. Although not statistically significant, also mitochondrial calcium content at 10 min of reperfusion tended to be lower in 0.5 Lac hearts compared to 0 Lac hearts (Figure 6B).

### Mitochondrial Calcium Retention Capacity

Mitochondria that were isolated from the non-ischemic control hearts were able to retain more calcium and take it up faster when challenged with 0.3 mM exogenous calcium (Mix 2) compared to the mitochondria that were isolated from the hearts from the four experimental (ischemic) groups (no statistical test performed as non-ischemic control hearts *n* = 4; Figures 6C,D). However, no differences in neither the calcium retention capacity nor the duration of maximal calcium uptake were observed between the four experimental groups.

## Discussion

Using an isolated rat heart model of DCD, we demonstrate that DCD-relevant levels of pre-ischemic lactate (1 mM) significantly reduce contractile recovery and increase cell death marker release during reperfusion compared to the physiologic pre-ischemic levels (0.5 mM). Although increased pre-ischemic lactate lowered oxidative stress and increased PGC-1α mRNA expression during reperfusion; this, alone, was insufficient to maintain functional recovery at DCD-relevant levels. Furthermore, in parallel with decreased function and greater cell death, cytochrome c (cyt c) release increased and calcium content tended to increase with 1 vs. 0.5 mM pre-ischemic lactate levels, indicating greater mitochondrial damage and suggesting a deleterious calcium overload in these hearts. This was supported by our finding that the inhibition of the mitochondrial calcium uniporter with Ru360 during early reperfusion improved the functional recovery and reduced the release of mitochondrial damage marker in hearts perfused with DCD-relevant pre-ischemic levels of 1 mM lactate, although a statistically significant decrease in the mitochondrial calcium content was not observed with the addition of Ru360.

Providing the heart with 0.5 or 1 mM lactate in addition to 11 mM glucose and 1.2 mM palmitate did not alter the contractile function during 20 min of aerobic perfusion (baseline) compared to the hearts without a pre-ischemic lactate. Samaja et al. showed that Langendorff perfusion of non-ischemic hearts with high levels of lactate (20 mM) vs. no lactate in KHB buffer did not affect the heart rate, but decreased the developed pressure (41). However, considering the relatively short aerobic perfusion time and moderate levels of lactate in our experimental conditions, no differences in the contractile function during baseline perfusion were expected.

Lactate levels in the (recirculating) perfusate during the pre-ischemic, aerobic perfusion tended to decrease in hearts with 0.5 or 1 mM lactate, indicating a net lactate use, whereas they were increasing in 0 mM lactate hearts, indicating a net lactate release. A study in cultured myotubes has shown that the acute addition of lactate inhibited glucose and oleic acid oxidation (42). Furthermore, lactate can inhibit mitochondrial β-oxidation by increasing acetyl-CoA and thereby malonyl-CoA formation, which inhibits carnitine-palmitoyl-transferase I (20). Therefore, in 0.5 and 1 Lac vs. 0 Lac hearts, proportionately more lactate is likely used/oxidized to generate ATP, permitting a similar cardiac function among groups prior to ischemia. When no lactate was added exogenously, glucose and palmitate were the only energy substrates. High levels of palmitate, as under our experimental conditions (1.2 mM), imply high rates of fatty acid oxidation, which is known to inhibit glucose metabolism. Inhibition of pyruvate dehydrogenase activity through fatty acid oxidation leads to a glycolysis-glucose oxidation mismatch and increased lactate production (43), in accordance with the net lactate release observed in 0 Lac hearts.

Lactate is slowly becoming recognized as a signaling molecule (21). In our hearts, after 60 min of reperfusion, the mRNA expression levels of PGC-1α were positively correlated with pre-ischemic lactate. PGC-1α is the master regulator of mitochondrial biogenesis, and there is a close relationship between PGC-1α and lactate (21). Not only does PGC-1α regulate lactate homeostasis by favoring lactate catabolism, but also lactate itself has been shown to upregulate PGC-1α gene expression (in mouse skeletal muscle) (21, 23). In rat skeletal muscle cells, in response to PGC-1α, the expression of MCT1, which facilitates lactate uptake, was suggested to increase (44). Correspondingly, we did not only find greater mRNA expression levels of PGC-1α, but also of MCT1, with increasing pre-ischemic lactate levels. Furthermore, rising lactate levels in the L6 cell culture have been shown to occur in parallel with increases in the MCT1 protein levels (45).

Carbonylated proteins at 60 min of reperfusion, a marker of oxidative stress damage, correlated negatively with the pre-ischemic lactate levels. This finding is in agreement with the positive correlation of PGC-1α with the pre-ischemic lactate levels, as PGC-1α, in addition to regulating mitochondrial biogenesis, plays a role in the stimulation of genes that modulate anti-oxidant defense systems (46). Tauffenberger et al. reported, in neuroblastoma cells, that lactate promoted a mild ROS burst which further triggered antioxidant defense systems and pro-survival pathways, ultimately to protect the cell against oxidative stress (47).

**Figure 7:**
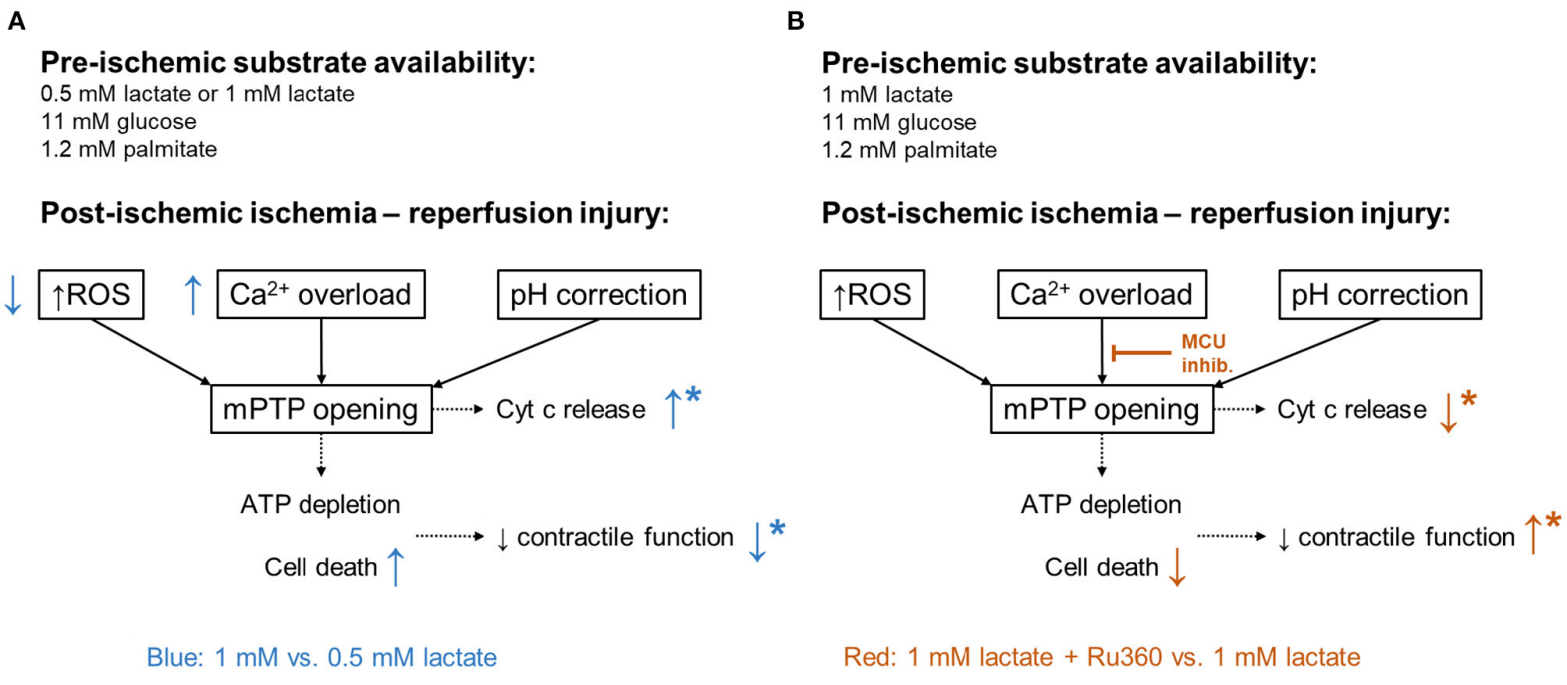
Mediators of ischemia-reperfusion injury. In black: major mediators of lethal reperfusion injury: reactive oxygen species (ROS), calcium (Ca^2+^) overload, and rapid pH correction contributing to the opening of the mitochondrial permeability transition pore (mPTP). ATP, adenosine triphosphate; Cyt c, cytochrome c. **(A)** In blue: differences observed in 1 Lac hearts compared to 0.5 Lac hearts. **(B)** In red: differences observed in 1 Lac + Ru360 hearts compared to 1 Lac hearts. *Indicates statistically significant difference; *p* < 0.05.

Post-ischemic cyt c release, a marker of mitochondrial damage, was significantly lower in the physiologic lactate group compared to hearts with no lactate or DCD-relevant levels of lactate. These results followed the observed pattern of post-ischemic functional recovery. Cyt c release marks the opening of mPTP which results in the uncoupling of oxidative phosphorylation leading to ATP depletion and cell death, and finally, to a reduced contractile function (24, 48). The different key mediators of ischemia-reperfusion injury leading to mPTP opening include mitochondrial calcium overload, oxidative stress, and rapid pH restoration (Figure 7) (24). However, as mentioned above, protein carbonylation (oxidative stress damage marker) was lowest in 1 mM lactate hearts, whereas in this experimental group, post-ischemic contractile function and cyt c release were worse compared to 0.5 mM lactate hearts. Therefore, lower oxidative stress, alone, was insufficient to preserve the mitochondrial integrity and to maintain functional recovery at DCD-relevant lactate levels. In contrast to oxidative stress, the pattern of calcium tissue content at 60 min of reperfusion corresponded to the results observed in cyt c and contractile function, indicating a role for calcium overload in the worse post-ischemic function in hearts with DCD-relevant lactate levels.

Through the inhibition of the MCU with Ru360 during early reperfusion in hearts with DCD-relevant pre-ischemic lactate levels, we found an improved post-ischemic recovery of LV work (Figure 7). Ru360 has already been shown to prevent hearts from irreversible injury after ischemia in isolated rat hearts (26). However, De Jesús Garcia-Rivas et al. firstly did not have any lactate in their perfusate and secondly, perfused their hearts before and after ischemia with Ru360 (26), while we demonstrate the protective effects with Ru360 added only at reperfusion. Nonetheless, cyt c release was significantly lower and total tissue calcium tended to be lower in 1 Lac hearts with vs. without Ru360, indicating better mitochondrial preservation. Investigations in MCU^−/−^ mice have shown that MCU-dependent calcium uptake is critical for the opening of mPTP in response to elevated cytosolic calcium concentrations and that specifically for calcium-induced mitochondrial permeability transition, MCU appears to be important (49).

Neither the calcium retention capacity nor the calcium content in the freshly isolated cardiac mitochondria after 10 min of reperfusion differed between the experimental groups. Importantly, post-ischemic functional differences between the groups occurred only after 20 min of reperfusion, while at 10 min, all functional parameters were similar, suggesting that this may be a reason why no differences in the mitochondria were measured at 10 min of reperfusion. Internal positive controls (non-ischemic control hearts) confirmed a satisfying quality for our mitochondria isolations with a substantially lower mitochondrial calcium content, faster calcium uptake, and longer retention capacity compared to our ischemic hearts. Lower mitochondrial calcium content was expected, but not observed, in the 1 mM lactate hearts with Ru360 compared to those without Ru360. Although Ru360 has been shown to specifically inhibit MCU and not ryanodine receptors or calcium cycling in the sarcoplasmic reticulum, an incomplete inhibition of MCU has been reported by De Jesús Garcia-Rivas et al. (26). Despite not significantly decreasing the mitochondrial calcium content after 10 min of reperfusion, we found that Ru360 preserved mitochondrial integrity and post-ischemic function after 60 min reperfusion in our experimental setting in hearts exposed to DCD-relevant levels of pre-ischemic lactate.

### Limitations

This study has several limitations. Perfusion with crystalloid buffer was chosen to strictly control energy substrate availability. However, perfusions with blood and longer reperfusion periods could increase the clinical relevance of our findings, as could studies in larger animal models. Furthermore, evaluation of infarct size, histological analyses, and mitochondrial respiration measurements could help to confirm our results.

## Conclusions

We investigated the effects of different levels of pre-ischemic lactate on post-ischemic contractile function and molecular mechanisms in an isolated rat heart model of DCD. Our findings that even slight modifications of 0.5 mM in pre-ischemic lactate levels influenced the cardiac post-ischemic recovery highlight the importance of substrate availability before ischemia. Lactate levels increase due to hypoxia before cardiac arrest in DCD Maastricht category III donors, and we highly recommend that the influence of energy-substrate availability should be considered in both the pre-clinical DCD models and in the optimization of the DCD heart procurement protocols. Furthermore, in DCD, interventions in the donor before death declaration and graft procurement are limited due to ethical and legal constraints, and these increasing levels of lactate cannot be avoided. Therefore, cardioprotective strategies applied at the onset of reperfusion that are tailored to minimize the lactate-potentiated ischemia-reperfusion damage hold a great potential. Our findings suggest that the inhibition of MCU with Ru360 at the onset of reperfusion in cardiac DCD grafts is a promising approach and should be further considered.

## Data Availability Statement

The raw data supporting the conclusions of this article will be made available by the authors, without undue reservation.

## Ethics Statement

The animal study was reviewed and approved by Ethics Committee for Animal Experimentation, Berne, Switzerland (Veterinärdienst des Kantons Bern).

## Author Contributions

MA: contributed to all aspects of this manuscript including study planning and design, method development, performing the experiments, data collection, analysis and interpretation, and preparation of the manuscript. AS: participated in study planning and design, performing the experiments, and data collection and analysis. SG, NM-C, and NKa: participated in method development, performing the experiments, and data collection. MS, RW, and NKe: participated in method development and experimental troubleshooting. TC: participated in data interpretation and preparation of the manuscript. SL: participated in study planning and design, method development, data analysis and interpretation, and preparation of the manuscript. All authors contributed to the article and approved the submitted version.

## Conflict of Interest

The authors declare that the research was conducted in the absence of any commercial or financial relationships that could be construed as a potential conflict of interest.

## Abbreviations

CCCP: carbonyl cyanide 3-chlorophenylhydrazone
CsA: cyclosporine A
Cyt c: cytochrome c
DCD: donation after circulatory death
dP/dt max: maximum first derivative of left ventricular pressure
dP/dt min: minimum first derivative of left ventricular pressure
H-FABP: heart-type fatty acid binding protein
HIF-1α: hypoxia-inducible factor-1 alpha
LV work: left ventricular work
MCT1: monocarboxylate transporter 1 (Slc16a1)
MCU: mitochondrial calcium uniporter
mPTP: mitochondrial permeability transition pore
O_2_C: cardiac oxygen consumption
PGC-1α: peroxisome proliferator–activated receptor gamma coactivator-1 alpha
RPLP0: ribosomal protein lateral stalk subunit P0
Ru360: oxygen-bridged dinuclear ruthenium amine complex
WLST: withdrawal of life-sustaining therapy
Ywhaz: tyrosine 3-monooxygenase/tryptophan 5-monooxygenase activation protein.
