# Supplementary material for: Pre-ischemic Lactate Levels Affect Post-ischemic Recovery in an Isolated Rat Heart Model of Donation After Circulatory Death (DCD)
Source: Front Cardiovasc Med. 2021 Jun 14;8:669205. doi: 10.3389/fcvm.2021.669205 (PMC8236508; doi:10.3389/fcvm.2021.669205)
Supplement: Supplementary file 1 [file Data_Sheet_1.PDF]

Supplementary Figures

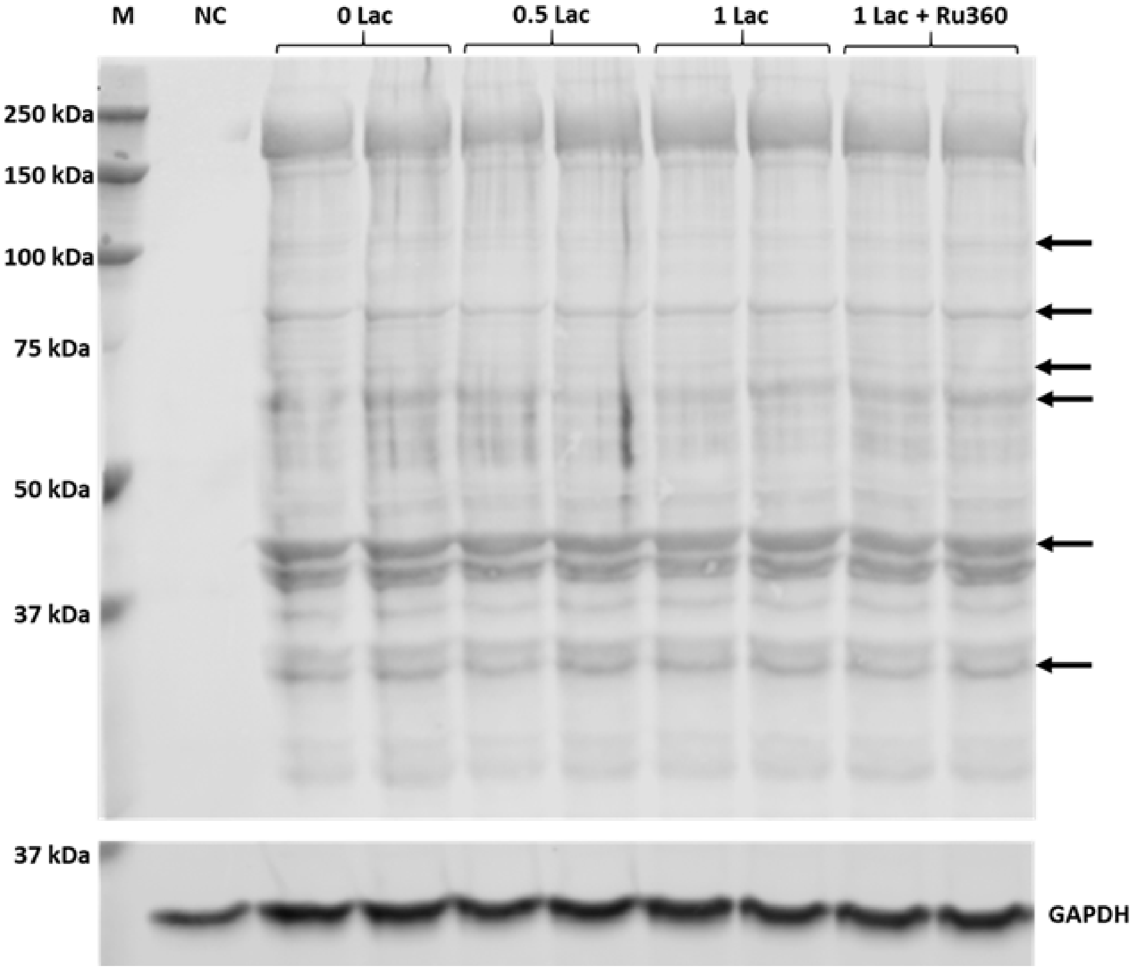

Figure S1. Protein carbonylation representative blot imaging. Total bars in Figure 5 refer to the densitometry values of the bands from size 100 kDa to 32 kDa. M, marker; NC, non-derivatized sample used as negative control; ← arrows indicate the bands analyzed.
