# Supplementary material for: Pre-ischemic Lactate Levels Affect Post-ischemic Recovery in an Isolated Rat Heart Model of Donation After Circulatory Death (DCD)
Source: Front Cardiovasc Med. 2021 Jun 14;8:669205. doi: 10.3389/fcvm.2021.669205 (PMC8236508; doi:10.3389/fcvm.2021.669205)
Supplement: Supplementary file 2 [file Data_Sheet_2.PDF]

Supplementary Figures

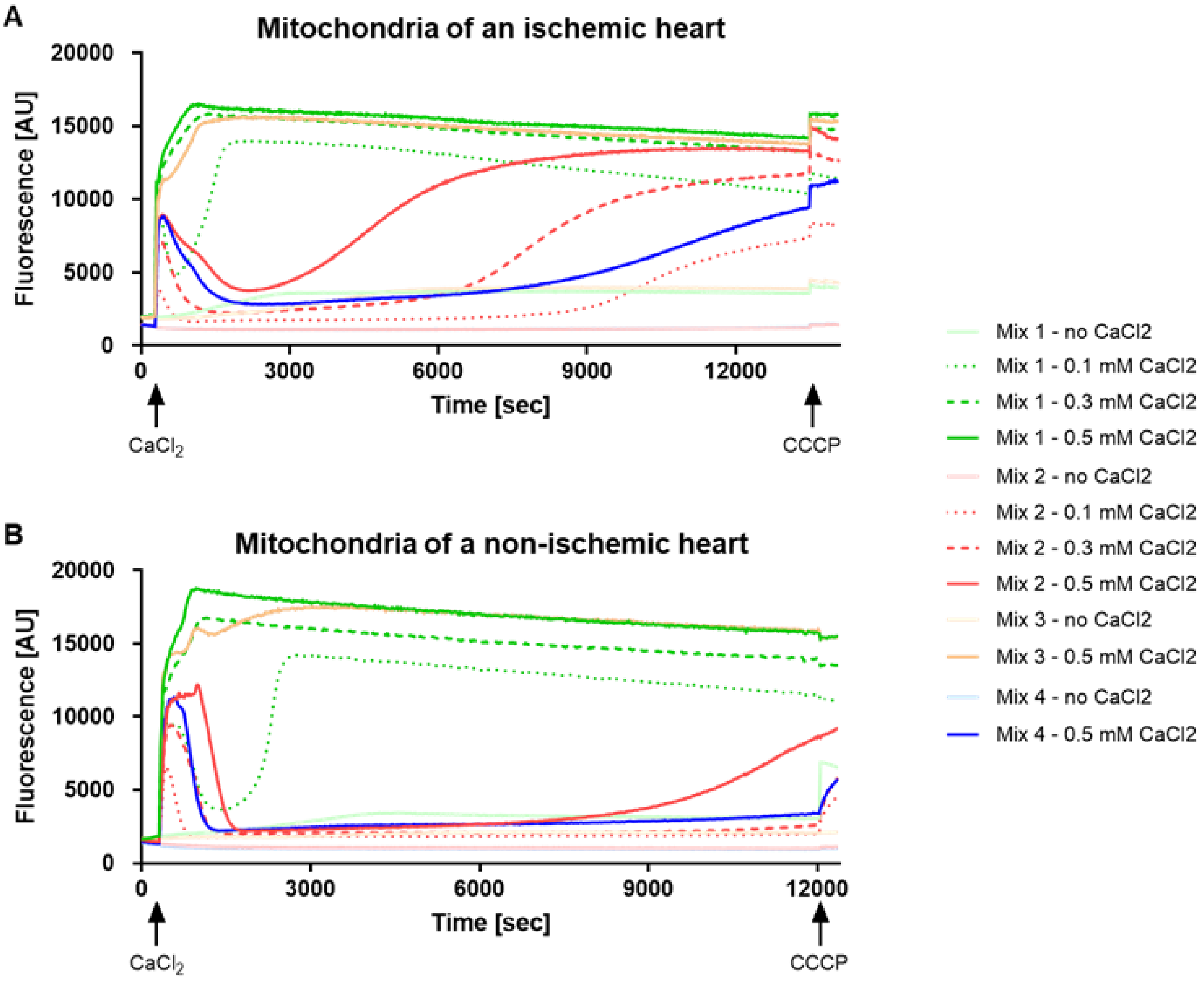

Figure S2. Calcium retention capacity dynamics. In example plots for calcium retention capacity, fluorescent values are represented from (**A**) isolated mitochondria from a heart that underwent 28.5 min global, warm ischemia and 10 min reperfusion, and from (**B**) isolated mitochondria from a heart that was perfused aerobically for one hour. After a baseline measurement of 3 min, the reaction was initiated through the addition of a single calcium spike (either 0.1, 0.3 or 0.5 mmol/L CaCl2) and fluorescence was monitored. At the end of the assay, 0.1 mmol/L carbonyl cyanide 3-chlorophenylhydrazone (CCCP) was added. Specific composition of the different mixes is explained in detail in Table 2.
